# ReFlexIn: A Flexible Receptor Protein-Ligand Docking Scheme Evaluated on HIV-1 Protease

**DOI:** 10.1371/journal.pone.0048008

**Published:** 2012-10-24

**Authors:** Simon Leis, Martin Zacharias

**Affiliations:** Technische Universität München, Physik-Department T38, Garching, Germany; Weizmann Institute of Science, Israel

## Abstract

For many targets of pharmaceutical importance conformational changes of the receptor protein are relevant during the ligand binding process. A new docking approach, ReFlexIn (Receptor Flexibility by Interpolation), that combines receptor flexibility with the computationally efficient potential grid representation of receptor molecules has been evaluated on the retroviral HIV-1 (Human Immunodeficiency Virus 1) protease system. An approximate inclusion of receptor flexibility is achieved by using interpolation between grid representations of individual receptor conformations. For the retroviral protease the method was tested on an ensemble of protease structures crystallized in the presence of different ligands and on a set of structures obtained from morphing between the unbound and a ligand-bound protease structure. Docking was performed on ligands known to bind to the protease and several non-binders. For the binders the ReFlexIn method yielded in almost all cases ligand placements in similar or closer agreement with experiment than docking to any of the ensemble members without degrading the discrimination with respect to non-binders. The improved docking performance compared to docking to rigid receptors allows for systematic virtual screening applications at very small additional computational cost.

## Introduction

Protein receptor molecules can undergo a variety of conformational changes upon complex formation with binding partners ranging from small side chain adjustments to global backbone conformational changes and even refolding of loop structures [1], [2], [3], [4], [5], [6], [7]. The importance of receptor flexibility for drug design has been extensively reviewed [8], [9], [10], [11], [12], [13], [14] and it has been shown that already small conformational changes of the receptor backbone in the range of 1Å can significantly affect receptor-ligand interactions [15].

Popular fast docking methods typically represent the protein receptor molecule as a potential grid assuming a rigid receptor structure during ligand-receptor docking [16]. In order to account for conformational changes of the receptor it is possible to represent the receptor by an ensemble of rigid structures and to either use each structure separately for sequential docking or switching between ensemble members employing a Monte Carlo (MC) approach [17], [18], [19], [20]. The structures for such ensemble can be derived either computationally, e.g. by molecular dynamics (MD) simulations or using appropriate structural modelling methods. Alternatively, the ensemble can also be comprised of different experimental structures or different homology models of a target receptor protein [21]. Instead of sequential docking, more sophisticated methods have also been suggested which include, for example, approaches that apply an ensemble average or select a consensus receptor out of the ensemble [22].

We have recently introduced a flexible receptor docking approach based on representing a flexible receptor by a series of potential grids each corresponding to one discrete receptor conformation. If neighboring receptor conformations do not deviate too strongly it is possible to approximately represent the potential of intermediate conformations (between two discrete states) by a linear interpolation between neighboring potential grids. In that way it is possible to effectively deform the receptor conformation continuously along a collective conformational degree of freedom. The method, termed “ReFlexIn” (Receptor Flexibility by Interpolation), was initially applied to the docking of several ligands to protein kinase A in the unbound (apo) from using structures deformed along the softest normal mode obtained from an elastic network model of the protein [23]. It yielded docked ligand-receptor complexes in very good agreement with experiment. In addition, the approach resulted in the selection of receptor conformations much closer to the ligand-bound (holo) PKA structure compared to the apo structure (although no information on the holo-structure was included). This was achieved at very modest additional computational cost compared to docking to a single rigid receptor structure and it is much faster than sequential docking to an ensemble of conformers.

**Figure 1:**
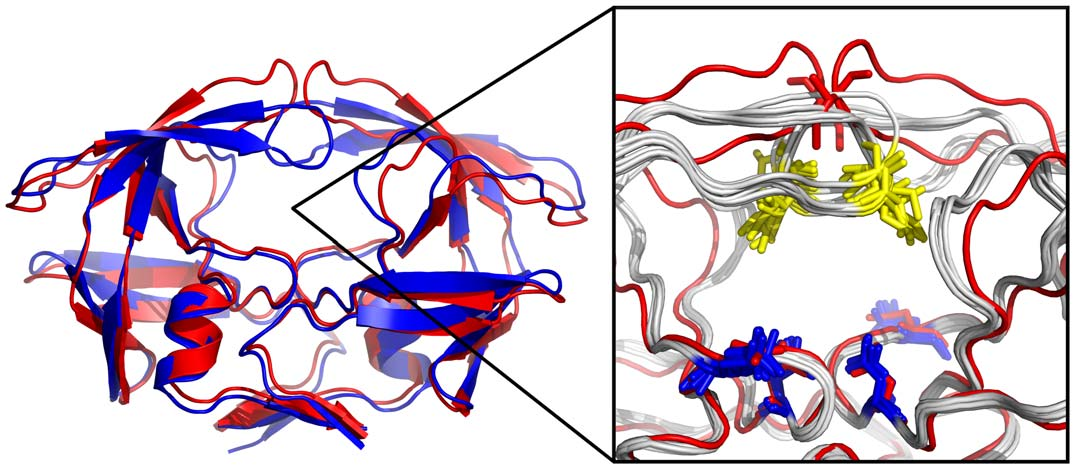
Conformational changes in HIV-1 Protease. Left: Cartoon representation of an unbound (red, pdb ID 3HVP) aligned to a bound receptor structure of HIV-1 protease (blue, pdb ID 7UPJ). Upon ligand binding, the closure of the two flaps narrowing the binding site is clearly visible. – Right: Close-up view of the binding site. Apo HIV-1 protease (red) aligned to 7 different bound forms of HIV-1 protease shown in grey (ligands not shown). Four flexible side chains (yellow: Ile'50 residues and blue: Asp'25 of both monomers of the homo-dimeric protease structure) at the substrate and inhibitor binding site are represented as stick structures (colored red in case of the apo structure).

The methodology is not restricted to deformations along a normal mode direction of a protein. In the present study it is extended to an in principle arbitrary set of receptor conformations that can be ordered according to some measure of structural similarity between neighboring conformations. The “reaction coordinate” for deformations is then represented by a hypothetical path connecting all structures in the ensemble. The interpolation scheme allows for a smooth interpolation between the “end-points” of the ensemble along the pathway.

**Figure 2:**
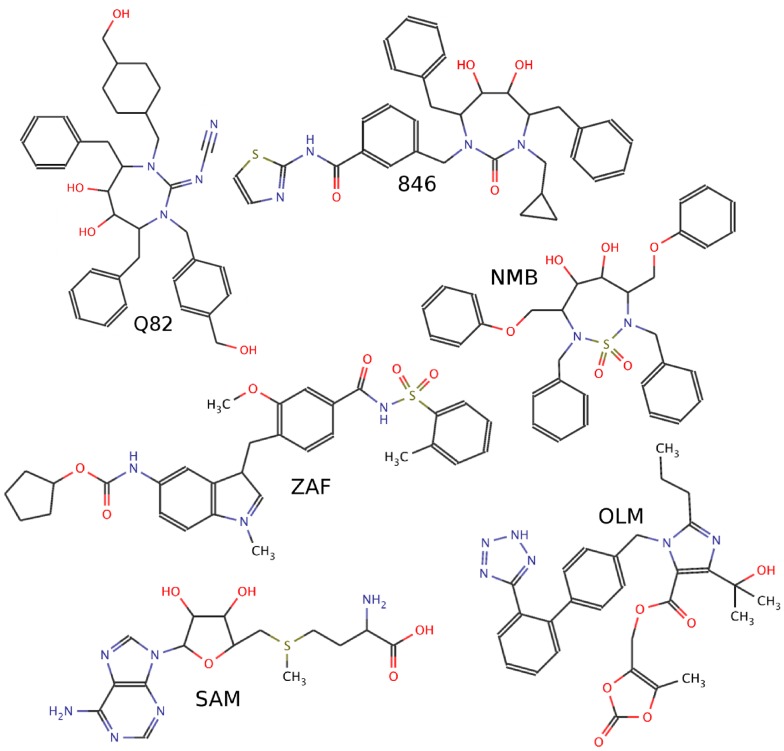
Examples for the used ligand test-sets. Sample chemical structures of HIV-1 protease binding ligands (binders: Q82, NMB and 846), and non-binders of similar size and composition (ZAF, OLM, SAM) used in the ligand test-set. The complete ligand test sets are listed in Figure S1.

The method has been applied to HIV-1 (Human Immunodeficiency Virus 1) protease, a member of the aspartyl protease class. It forms a homodimer which plays a crucial role in the HIV replication cycle and is therefore an interesting and well-studied drug target. Its specific role is the cleavage of newly synthesized HIV poly-proteins into functional proteins that are required for an active HIV virion. Inhibiting drug molecules that mimic a polypeptide chain bind tightly to the protease active site and can block the protein's function, thus preventing the HI-virus from maturation [24]. Possible inhibitors bind at the active site that is located in the central part of the homodimer [25]. The tunnel-like binding site is covered by two *beta*-sheet flaps that have been shown to be open in the un-liganded HIV-1 protease and take a closed conformation upon inhibitor binding [26], [27]. The first experimental structure of HIV-1 protease has been solved in 1989 [28] and since then, various 3D-structures of bound and unbound states have been deposited in the protein data base. Since the HIV-1 protease structure undergoes significant conformational changes upon ligand binding, it represents a challenging target for docking approaches. While the flexibility of protease inhibitors has been typically included in different computational docking studies [29], [30], [31], [32], [33], the efficient and accurate consideration of the protease flexibility is still a challenging task. It is also possible to combine molecular dynamics (MD) simulations with molecular docking to predict ligand-receptor interactions treating both the ligand and the receptor conformation flexible. Such approach has been applied to the HIV-1 protease system [34]. However, since it is necessary to run one or several computationally demanding MD simulations for each putative ligand candidate, the approach is prohibitively expensive if one needs to screen hundreds or even thousands of putative drug candidates.

**Figure 3:**
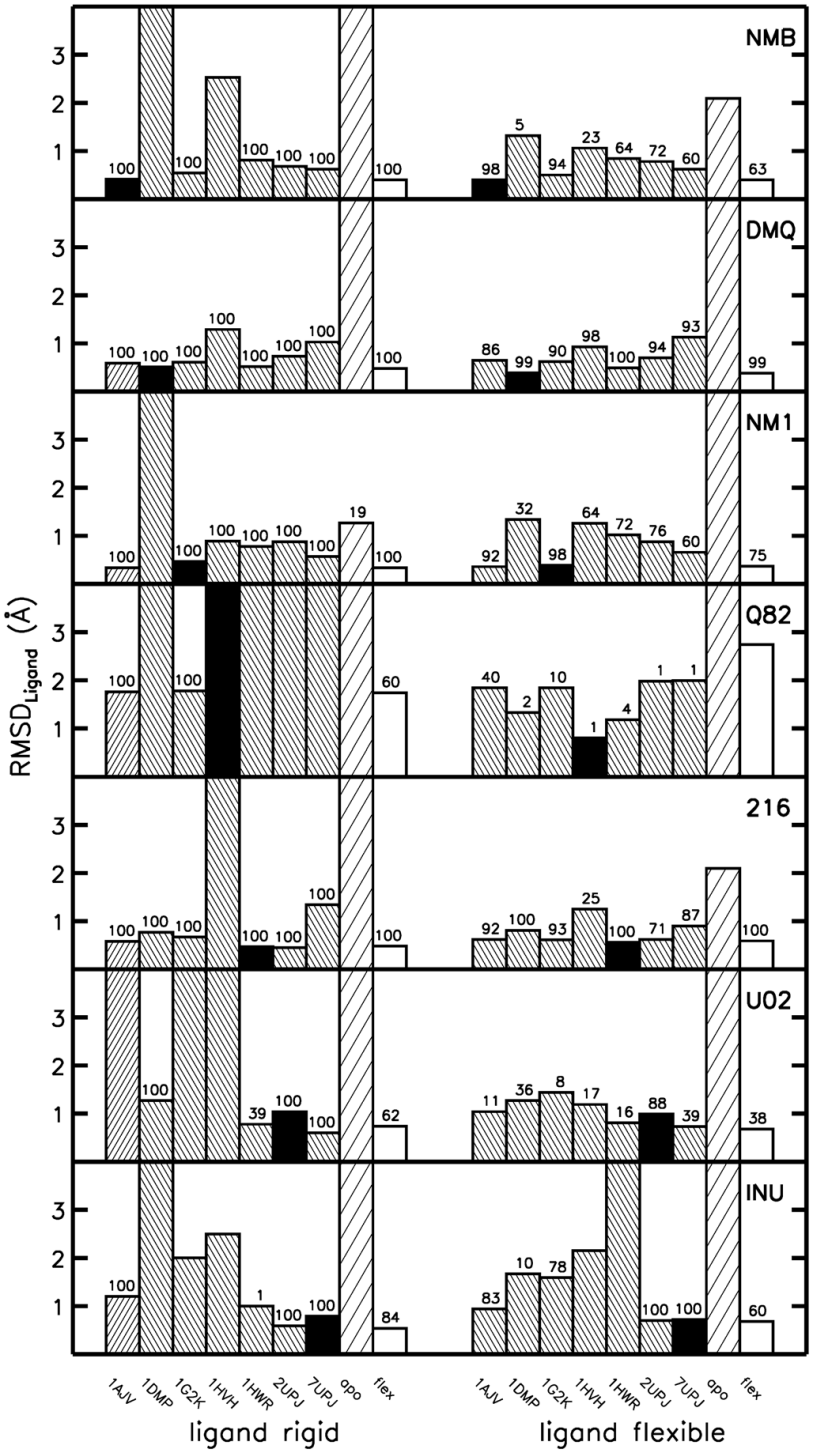
True binder test-set docking results of cross docking, rigid, and flexible receptor docking. Docking results in terms of deviation of the docked ligand from the native placement for the rigid receptor cross docking, apo docking, and flexible receptor docking of the HIV-1 protease true binder test set. Bar height indicates the best RMSD solution found in 100 separate dockings (values are cut at 4Å). Narrow shaded bars are the dockings of the ligand denoted in the upper right corner of each plot into the rigid receptor structure as indicated in the bottom. Wide shaded bars show the results of the rigid receptor apo docking, non-shaded bars for the flexible receptor docking. Numbers on top of bars indicate the number of dockings per 100 separate docking runs that yield ligand RMSD values below 2Å. Holo docking results (docking the ligand back to the receptor structure from which it was extracted) are shown as filled bars.

Huang et al. introduced an ensemble docking method that allows simultaneous optimization of ligand placement and receptor conformation out of a set of protein structures and found significant improvement compared to docking to single structures [22]. This method was successfully applied by the same authors to an ensemble of NMR (nuclear magnetic resonance)-derived structures of HIV-1 protease [35]. The application of ensembles of HIV-1 protease crystal structures and from MD simulations has also been shown to improve docking results [36].

**Figure 4:**
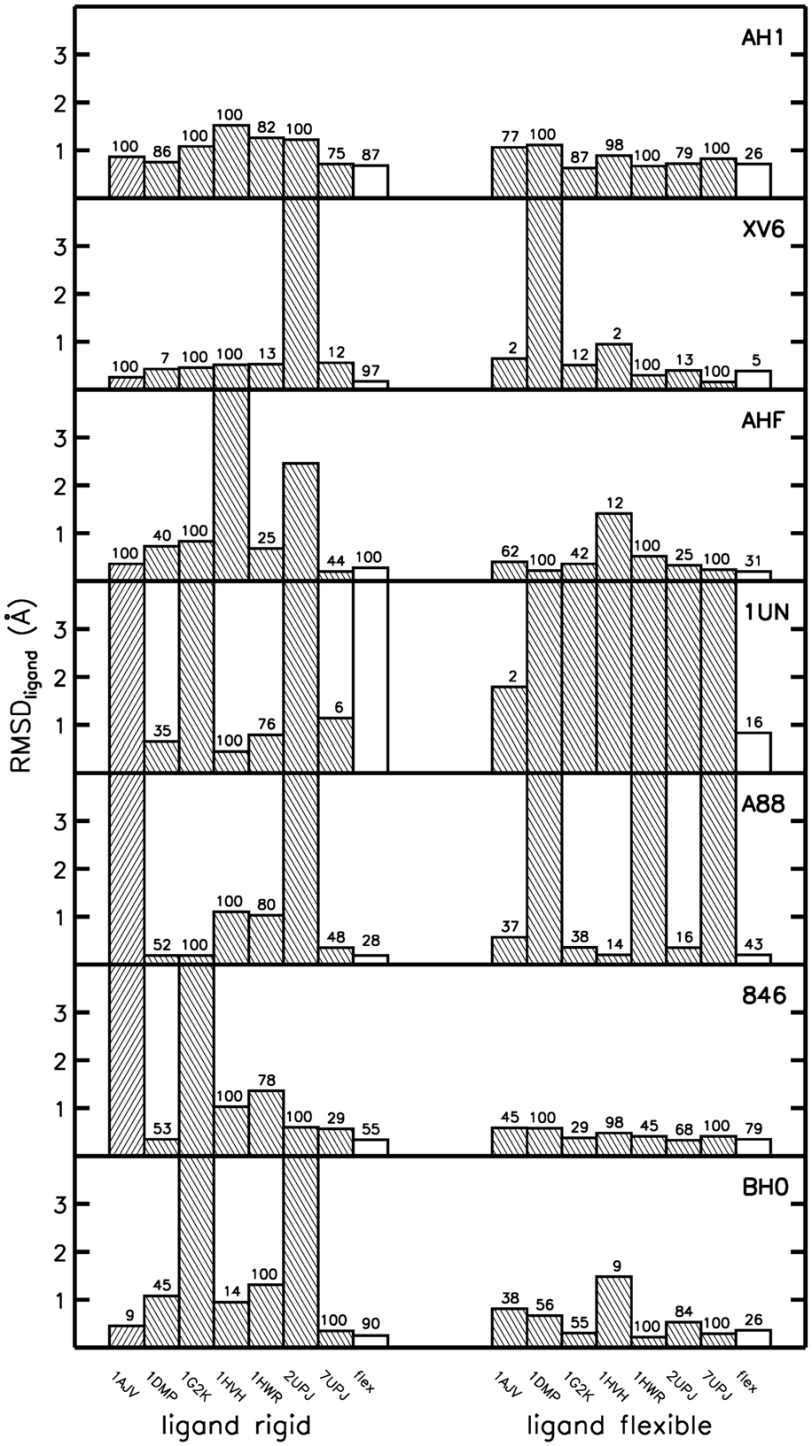
Foreign binder test-set docking results of cross docking, rigid, and flexible receptor docking. Same as Figure 3 (see legend of Figure 3) for a second set of ligands known to bind to HIV-1 protease (the foreign binder ligand test-set).

In the present study the ReFlexIn receptor-ligand docking approach was used for docking a series of putative ligands to HIV-1 protease into an ensemble of different bound (holo) conformations as well as to a set of structural models obtained by morphing the protease apo structure towards one bound structure. In contrast to other ensemble based docking methods it allows the smooth and continuous deformation of a receptor at very small additional computational demand compared to docking to a rigid receptor. On a set of ligands known to bind to HIV-1 protease and a set of non-binding ligands of similar size and physico-chemical properties, the docking methodology resulted in more accurate prediction of ligand binding placement compared to docking to a single receptor in a bound conformation without loss in the discrimination between known binders and non-binding ligands. The results indicate that the approach could be well suited for systematic docking screening of a large number of putative ligands for a given flexible receptor target.

**Figure 5:**
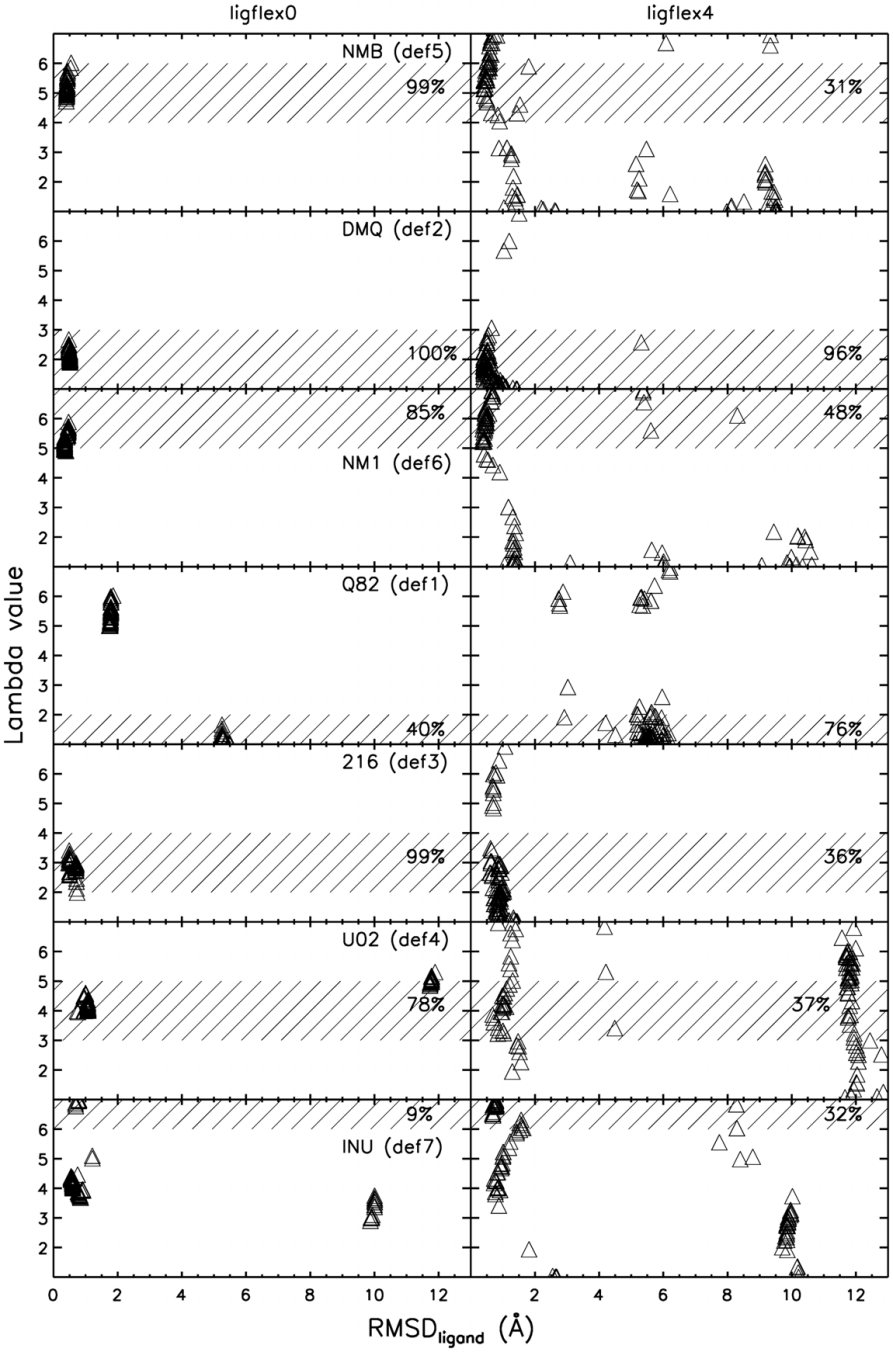
Ligand placement quality vs. docked receptor structure. HIV-1 protease structure vs. RMSD_ligand_ for flexible receptor docking applied to the true binder test set. Variation of the parameter lambda corresponds to a continuous deformation of the HIV-1 protease receptor structure beginning at the bound form pdb ID 1HVH (lambda = 1) and following the order 1DMP, 1HWR, 2UPJ, 1AJV, 1G2K and ending with 7UPJ (lambda = 7) which represent a minimum RMSD_protein_ pathway. Ligand RMSD corresponds to the deviation of the 100 docked ligand coordinates from the native structure after best superposition of the receptor structure. Correlation between ligand placement and receptor structure are shown for both treating the ligand as rigid bound conformer or flexible (allowing bond rotation). The shaded areas are drawn to indicate the expected receptor deformation close to the conformation found in the crystal structure of the ligand in complex with HIV-1 protease. Numbers inside the shaded area give the percentage of correctly assigned lambda value for all 100 dockings.

## Materials and Methods

The AutoDock 4 docking program [37], [38] employs a grid representation for all interaction potentials with ligand atoms. Prior to the actual docking procedure, the auxiliary program AutoGrid pre-computes regular 3D potential grids with a user-defined spacing and position at the ligand binding site of the receptor. To each grid point, it assigns the calculated interaction energies for each ligand atom type, as well as the desolvation and electrostatic potential energy. Interactions between a receptor and a ligand atom can then be calculated from the eight nearest grid points by using a tri-linear interpolation and a lookup function scheme. All docking runs were performed using AutoDock's Lamarckian genetic algorithm (LGA) which employs variables (so-called genes) for translational, rotational, and torsion angle variation of the ligand.

**Figure 6:**
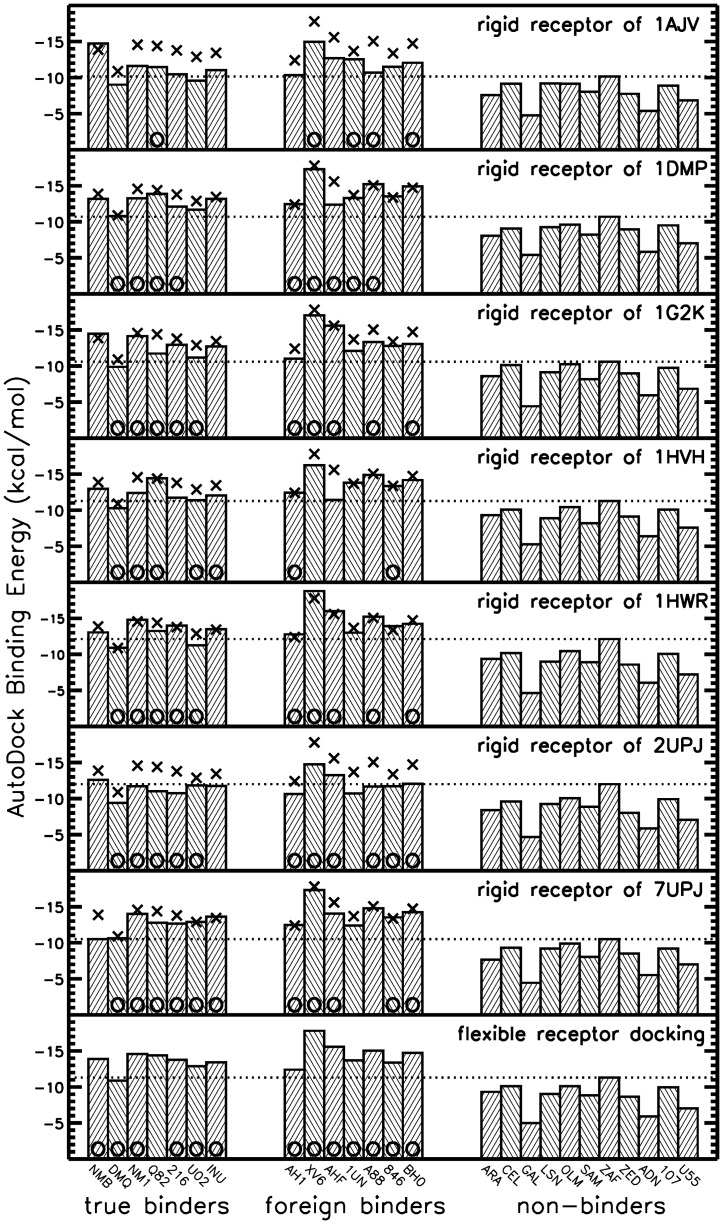
Binder non-binder discrimination by scoring energies. Best scoring results for docking of true, foreign and non-binders upon docking to 7 different rigid receptor structures and using flexible receptor docking (using interpolation between the 7 receptor structures). The dotted line marks the best binding energy obtained for the non-binder molecules (corresponds to the limit for distinguishing binders from non-binders). The cross symbols indicate the results of flexible receptor docking and are included for comparison for each docking to a rigid protease receptor (upper 7 rows). The O symbols are present at the bottom of each bar, if the respective best energy docking solution also yields an RMSD_ligand_ <2Å.

To include receptor flexibility along a set of different receptor structures, grid potential maps are calculated for a each receptor input structure (in the present study, seven receptor structures are representing either different bound forms of HIV-1 protease (see below) or intermediates of morphing between the apo and one holo form of HIV-1 protease). An additional conformational variable/gene, termed *lambda*, was integrated into the genetic algorithm. Lambda can (in the present case) take values between 1.0 and 7.0 each representing the lower and upper bound of receptor flexibility, respectively. During the docking search of the genetic algorithm, the lambda variable can be freely mutated (between 1.0 and 7.0), thus allowing for a variation of the receptor potential. This variation is not only discrete but continuous: non-integer values for lambda trigger a linear interpolation between the two nearest potential grids.

**Figure 7:**
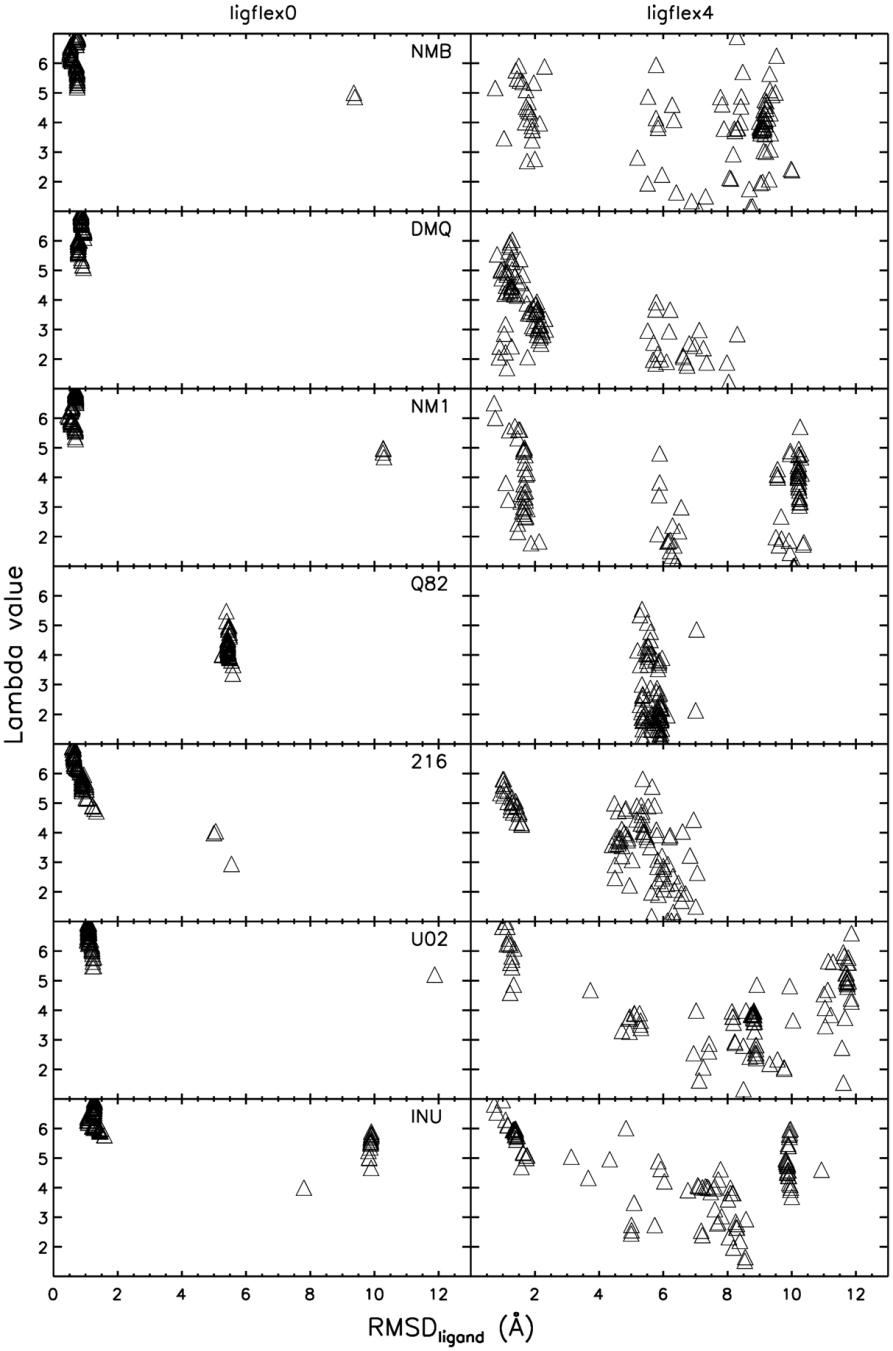
Ligand placement quality vs. docked receptor structure for the morphing approach. HIV-1 protease structure vs. RMSD_ligand_ for flexible receptor docking applied to the true binder test set. Variation of the parameter lambda corresponded to a continuous morphing of the HIV-1 protease receptor structure beginning at the apo form (pdb 3HJV, lambda = 1) and ending with one bound form (pdb2UPJ, lambda = 7). RMSD_ligand_ corresponds to the deviation of the 100 docked ligand coordinates from the native structure after best superposition of the receptor structure. Correlation between ligand placement and receptor structure are shown for both treating the ligand as rigid bound conformer of flexible (allowing bond rotation).

Receptor and ligand structures were prepared using the standard AutoDock preparation scripts and for both rigid and flexible receptor docking, the program version 4.2.3 was used with the same standard settings (grid calculations with 50×50×50 grid points of 0.375Å spacing centred at the HIV-1 Protease binding site). The Lamarckian genetic algorithm was used for 100 separate docking runs with the following settings: ga_pop_size = 150, ga_num_evals = 2,500,000, ga_num_generations = 27,000, ga_elitism = 1, ga_mutation_rate = 0.02, ga_crosover_rate = 0.8.

**Figure 8:**
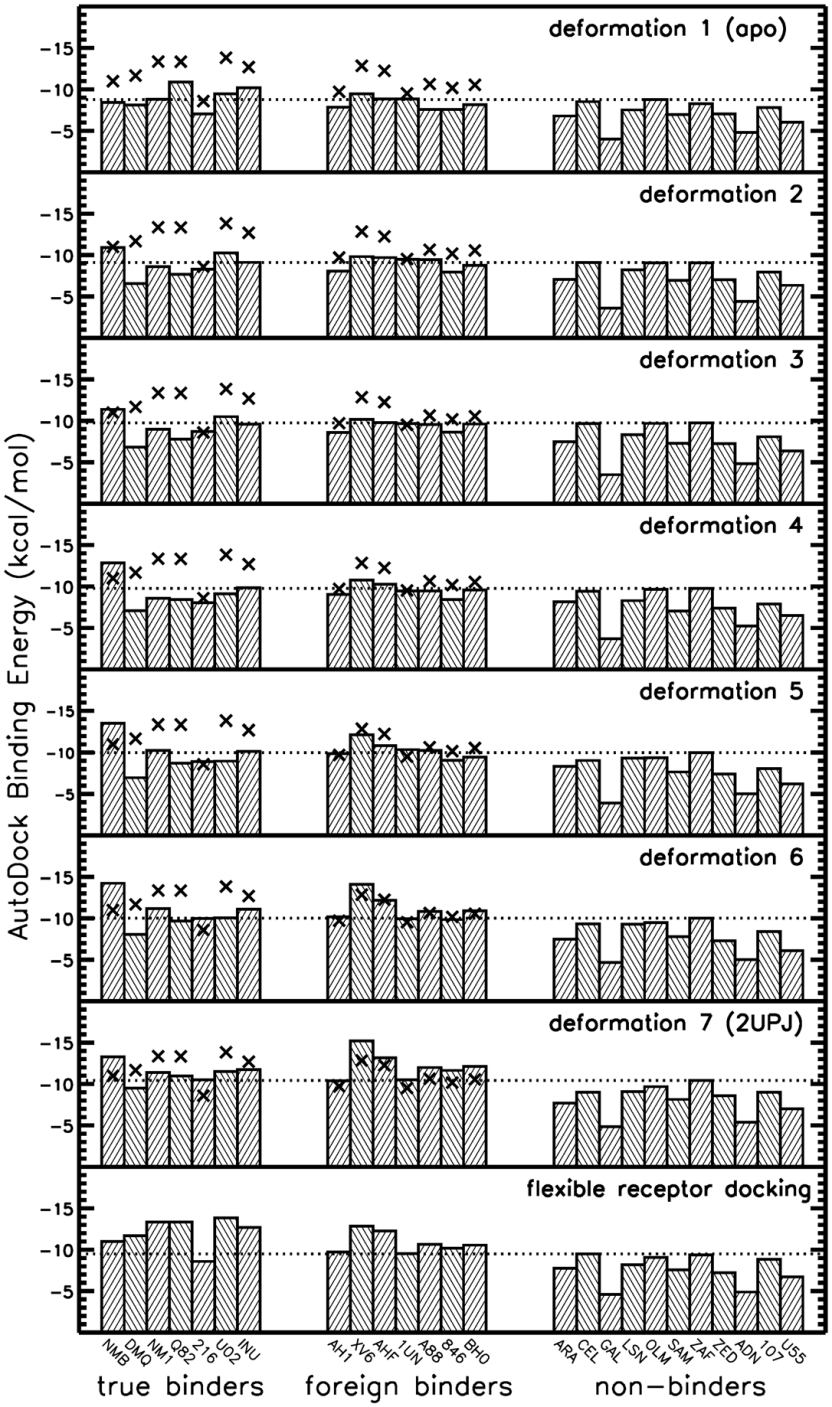
Binder non-binder discrimination by scoring energies for morphing approach. Best scoring results for docking of true, foreign and non-binders to apo (first row), 5 morphing intermediate receptor structures (row 2 to 6) and the bound receptor structure pdb ID 2UPJ and using flexible receptor docking (using interpolation between the 7 different receptor structures). The dotted line marks the best binding energy obtained for the non-binder molecules. The cross symbols indicate the results of flexible receptor docking and are included for comparison for each docking to a rigid protease receptor (see also legend of Figure 6).

The seven selected HIV-1 protease structures were extracted from pdb-files 1AJV, 1DMP, 1G2K, 1HVH, 1HWR, 2UPJ, and 7UPJ crystallized in the presence of different ligands each representing a different binding site structure (conformational changes are illustrated in Figure 1). *True binders* are ligands that are taken from the crystal receptor structures that are actually within the ensemble that is used for the flexible receptor docking. *Foreign binders* are binding ligands for which crystal structures in complex with HIV-1 protease are also known (AH1 from pdb ID:1AJX, ligand XV6 from pdb ID:1BV9, ligand AHF from pdb ID:1G35, ligand 1UN from pdb ID:1OHR, ligand A88 from pdb ID:1PRO, ligand 846 from pdb ID:1QBU, and ligand BH0 from pdb ID:1T7K). The term “foreign” refers to the fact that those ligands are extracted from bound HIV-1 protease structures with different binding site conformations compared to the structures that are included in the flexibility ensemble. *Non-binders* are molecules that do not bind to HIV-1 protease but that are of similar size and contain similar chemical groups and atom types as binders (ARA, CEL, GAL, LSN, OLM, SAM, ZAF, ZED, ADN, 107, U55). The first 8 non-binder molecules are taken from a previously employed ligand set for the evaluation of a rescoring scheme for the docking to HIV-1 protease [39]. The last three non-binders correspond to ligands that bind to protein kinases (ligands from pdb ID's 1FMO, 1FVV, and 1JSV). All ligands were docked either rigidly (in the configuration taken from the respective crystal structure) or with full flexibility of 4 flexible torsion angles per ligand. Recent studies have shown that ligand flexibility in AutoDock (as well as other popular docking algorithms as GLIDE, DOCK, or FlexX) should not exceed 6–8 flexible torsions to yield reasonable results [33], [38], [40]. Several examples of this study's test-set are shown in Figure 2, the complete ligand sets are shown in Figure S1.

### Ordering of the bound structures

The interpolation routine of grids representing neighboring conformations requires to order the receptor structures to minimize the deviation between neighboring receptor conformations. RMSD_Protein_ values were calculated between each possible set of two bound structures of the ensemble using only the atoms in a radius of 13Å around the binding site. All possible permutations to form a pathway going through the seven structures (this equals 7! = 5040) were examined for their total sum of RMSD differences and finally, the shortest path that includes the sequence with the smallest possible sum of RMSDs has been determined. For a set of 7 structures evaluation of all possible pathways through the structures is still possible. However, for a larger set of structures computationally more efficient dynamic programming methods could be used instead for finding an optimal path through all receptor conformations. The selected order - called deformation 1 to deformation 7 - is: 1HVH-1DMP-1HWR-2UPJ-1AJV-1G2K-7UPJ. Note, that the linear interpolation between the potentials of neighboring receptor structures along the path is likely to be most accurate if the selected neighbors along the path have the smallest possible pairwise RMSD. However, it should be noted that the choice with the smallest sum of RMSDs between neighboring receptor structures is not the only possible choice. For example, ordering based separately on global and/or local structural differences could be used and will be explored in future studies. Due to the lack of a reference crystal structure of those ligands in a 'foreign' bound form of the protease, the calculation of the ligand RMSD (RMSD_ligand_) is done as follows: All receptor structures including their ligands have been aligned to one common protease form (apo HIV-1 protease, pdb ID 3HVP), taking into consideration only the 13Å area around the binding site for alignment. For calculation of the RMSD_ligand_ values, the ligand positions from the aligned structures have been taken as reference for the correct solution.

## Results and Discussion

### Rigid Receptor Docking

The ReFlexIn approach [23] implemented in AutoDock represents receptor flexibility by interpolation between potential grids each representing a different conformation of the protein. This allows a smooth continuous deformation of a receptor structure along a selected conformational coordinate and the computational demand grows by only ∼50% compared to docking to a single rigid receptor structure. For the HIV-1 protease case, an ordered set of 7 different conformations each taken from crystal structures in complex with different ligands was used (Figure 1). The structures were ordered such as to minimize the pairwise RMSD of the ligand binding pocket between neighbors in the list (see Methods part). Docking was performed with a set of ligands known to bind to HIV-1 protease (binders, see also Figure 2) and a set of ligands of similar size and chemical composition known not to bind (non-binders, see also Figure 2 and Figure S1). The set of binders consisted of the 7 ligands taken from the complexes that were also used to form the ensemble of receptor structures (termed true binders) and an additional set of binders from other HIV-1 protease complexes (termed foreign binders, see Methods).

In order to compare the docking approach with rigid docking to individual HIV-1 protease structures, we performed systematic docking of all ligands to each of the 7 selected bound HIV-1 protease structures. In each case the ligand was treated either as rigid in the bound conformation of the corresponding crystal structure or as flexible allowing bond rotations around mobile bonds (see Methods for details). For each docking case, 100 separate docking runs with different initial conditions were performed to obtain statistically meaningful results (not all docking runs yielded the same final structure because AutoDock performs a stochastic genetic algorithm search). Docking of ligands (true binders) to its “own” bound receptor structure (holo docking to the experimental receptor conformation in complex with the same ligand) yielded in all cases a complex close to the native complex geometry (see Figure 3). Here, the threshold value for a correct ligand position is an RMSD_ligand_ below 2Å towards the native ligand position in the crystal structure. Note, that in most cases the docking solution with lowest RMSD also corresponded to the best scoring solution (see Table S2). An analysis of the best-scoring solutions comparing binders and non-binders is given below (in paragraph “Comparsion of binders and non-binders”).

Interestingly, cross docking of true and foreign binders to one of the 7 (rigid) bound receptor conformations yielded in several cases also docking placements in close agreement with experiment (often also as best scoring result, Figure 3 and 4). However, inspection of Figure 3 and 4 indicates that there are also several cases where cross docking fails. On average the docking results (in terms of best RMSD_ligand_) are better if the ligand is flexible (allowing bond rotation around a selected set of bonds, see Methods). Not unexpectedly, docking to the apo HIV-1 protease structure yields in most cases (except one, Figure 3) poor results (RMSD_ligand_ *≥*2Å) both for docking rigid or flexible ligands. Only for the NM1 inhibitor taken from the 1G2K HIV-1 protease structure in 19 of 100 dockings a ligand placement with RMSD below 2Å was found. The rest of the docked ligands preferentially bound in other cavities of the more open apo HIV-1 protease active site conformation.

ReFlexIn yields in most cases ligand placements that show the same or even lower deviations (RMSD_ligand_) compared with the best single receptor structure of the ensemble (see Figure 3 and 4). In case of ligand 1UN, a ligand placement flipped by 180° was always found as the best docking solution. This corresponds to a docking orientation with the nearly symmetric ligand rotated by 180° with respect to the quasi-symmetry axis but one functional group placed incorrectly relative to the native placement. Instead of using foreign ligands to test the method it is also possible to dock the true ligands to an ensemble of six receptor structures in ReFlexIn and leaving out the receptor that corresponds to the selected ligand (“leave-correct-structure-out”). As indicated in Table S1 the docking performance overall only slightly degrades with respect to inclusion of the receptor structure that corresponds to the docked ligand and is similar to the docking of foreign binders to the selected set of HIV-1 protease structures.

### Receptor structure deformation

In addition to the predicted ligand placement and scoring, the ReFlexIn approach also returns the conformation of the receptor structure, in the present case, along the series of different bound HIV-1 protease conformations. The value of the receptor conformation variable lambda indicates into which bound structure (or intermediate between bound structures for non-integer values of lambda), the ligand was finally docked. The performance to select near native receptor conformations was tested for the true binders. In case of using a rigid ligand, a clear correlation between ligand placement and receptor deformation was observed (Figure 5). Those docking solution which resulted in a near native placement of the docked ligand also selected a receptor “deformation” close (but often not exactly) to the native bound structure corresponding to the particular ligand (Figure 5). In those cases, the ligand was docked into a receptor structure that is geometrically very similar to the true receptor structure, thus the algorithm correctly selects a structure close to native out of the ensemble. In case of docking a flexible ligand such correlation is also apparent but weaker compared to the case of using rigid ligands (Figure 5). Only docking of ligand Q82 resulted in near native placements in combination with lambda values between 5 and 6 that deviate from the corresponding native bound receptor structure. Near native bound receptor structures were selected in combination with an incorrect placement of the ligand with an RMSD_ligand_ of ∼6Å. This result agrees with the cross docking searches (Figure 3) to single bound receptor structures where the docking of ligand Q82 into the receptor structures 5 and 6 (1AJV and 1G2K) yielded the best results.

### Comparison of docking binders versus non-binders

To test the algorithms' potential to distinguish between binders and non-binders, a test set of eleven non-binders was selected (ARA, CEL, GAL, LSN, OLM, SAM, ZAF, ZED, ADN, 107, U55) with similar size and chemical composition to binders. All ligands were docked flexible with 4 rotatable bonds per ligand. The best AutoDock binding energies from 100 separate flexible receptor docking runs of several flexible ligands (binders and non-binders) are shown in Figure 6. On average the binding score of binders (true and foreign binders) is more favorable than for the set of non-binders (by ∼5kcal/mol) upon docking to one of the 7 rigid bound receptor structures. However, some of the non-binders scores are almost as favorable as some of the binders (depending on which bound HIV-1 protease structure was used as receptor). The ReFlexIn docking gave results that are better than most of the docking results selecting a single receptor conformation and are close to the results for the best performing bound receptor structure (pdb ID 1HWR). The average score for the non-binders is only slightly more favorable compared to the best performing bound receptor structure. It indicates that inclusion of receptor flexibility does not significantly degrade the selectivity for binders vs. non-binders during docking. Moreover, for the ReFlexIn runs the best scoring placement also corresponded to the near-native placement with lowest RMSD_ligand_ (boxes with circles in Figure 5) except for ligand Q82.

### Morphing between bound and unbound structures

Instead of deforming a protein structure along a soft normal mode direction [23] or representation by a set of bound structures it is also possible to generate putative intermediate structures by morphing between an unbound (apo) and a bound protein structure. This can be particularly useful if there is only one bound and one unbound receptor protein structure available. It offers the possibility to not only identify ligands that bind to a bound form but also to possible intermediate structures on the path from an apo form to the holo receptor form. Even if only a limited set of bound receptor structures is available a morphing approach could be used to generate a sufficient set of putative sterically possible intermediates.

For the HIV-1 protease test case the input for the morphing was the apo structure (pdb ID 3HJV) and one bound structure (pdb ID 2UPJ). Five putative intermediate structures were created using the linear corkscrew morphing approach of the UCSF Chimera program [41]. To remove possible atomic overlaps and resulting unphysical potential energies during the morphing process, a short energy minimization for each of the generated intermediate steps was applied (deformation 1 for the most opened (the apo) state and deformation 7 for the bound protein structure).

Both for using rigid or flexible ligands, the flexible receptor docking identified ligand placements close to the native placement as best scoring solutions. With the exception of ligand Q82, RMSD_ligand_ values well below 2Å were obtained. The ReFlexIn docking clearly outperforms the docking to the rigid apo receptor. Also, a correlation between receptor deformation (towards the bound from, represented by lambda = 7) and near-native ligand placement was observed (Figure 7). This correlation is less pronounced in case of using flexible ligands compared to using a rigid ligand, indicating that successful docking to putative intermediate structures is already possible if conformational adaptation of the ligand is allowed.

### Docking of binders versus non-binders to the morphing ensemble

The representation of receptor flexibility in terms of morphed structures (between apo structure and the 2UPJ bound structure) was also used for comparing docking of known binders and non-binders (same set as used for docking to the set formed by a series of bound HIV-1 protease structures, see above). The ReFlexIn approach was compared to docking to single rigid morphing intermediates including the end-points: apo-structure and 2UPJ (Figure 8). On average the (best) scoring of known binders is slightly better than the scoring of non-binders. However, as expected, the selectivity for discriminating between non-binders and binders (difference in scoring of binders vs. non-binders) is for the apo form and each intermediate structure worse than in case of docking to any of the 7 bound structures (compare Figure 8 and Figure 6). Interestingly, the ReFlexIn approach performs overall slightly better than docking of the various binders and non-binders to the one bound receptor structure which forms one end-point of the ensemble of morphed structures (compare last and before last row of plots in Figure 8). This indicates that small putative adjustments of the receptor structure around the single bound conformation can help to improve the scoring of ligands and to also slightly improve the discrimination with respect to non-binders.

It should be emphasized that the primary benefit of using an ensemble of morphed structure for docking is the possibility to select ligands that may prefer to bind to possible intermediate structures in between apo and bound forms of the receptor structure. Since no HIV-1 protease structures with bound ligands trapped in some intermediate structure are available it is not possible to test the ability of the approach to identify such complexes. However, as shown in the application on known binders and non-binders, the inclusion of the information on putative intermediate structures appears not to degrade the ability of the approach to identify correct binding modes (for ligand placement and receptor structure) and the possibility to discriminate between binders and non-binders.

## Conclusions

The ReFlexIn approach implemented in the AutoDock 4 software has been applied to docking of various binders and non-binding ligands to HIV-1 protease. Receptor flexibility was represented by a continuous deformation between various ligand bound protease conformations or by a set of conformations obtained from morphing between the apo protease and one bound protease conformation. Ensemble docking applied to the HIV-1 protease has been shown to improve docking results compared to docking to single receptor conformations [35], [36]. However, these studies employed individual discrete structures during docking. Molecular dynamics based docking has also been successfully applied to the HIV-1 protease system [34]. Although this approach can in principle include all atomic degrees of freedom the result may depend on the starting configuration, simulation length and scoring scheme. In approaches that represent the receptor as potential grid many more ligand placements can be evaluated and consequently a more systematic evaluation is possible. In contrast to other ensemble docking approaches the ReFlexIn method allows for a smooth and continuous deformation of the receptor structure along a series of snapshots by interpolation between potential grids for each snapshot. In case of representing receptor flexibility by a set of bound protease structures the approach performed as good as or even better than using the best performing single rigid bound receptor. This indicates that the docking performance can profit from the procedure interpolating between discrete receptor structures. Also, since a best performing bound structure is not known in advance it is beneficial to use the ReFlexIn method instead of docking to each individual receptor structure.

In case of docking to a set of structures obtained from the morphing approach the ReFlexIn approach performed much better than docking to the apo form alone but even slightly better in terms of ligand placement and discrimination between binders and non-binders compared to docking to the one bound structure included in the morphing approach. In addition to identifying ligand binding to structures close to the bound receptor structure the latter approach allows in principle the simultaneous identification of ligands that may bind to putative intermediate structures in between apo- and holo receptor structures. This comes at rather modest additional computational demand which is only ∼50% higher compared to docking to single rigid receptor.

## Supporting Information

Figure S1 **Full ligand test-set containing 7 true binders NMB, DMQ, NM1, Q82, 216, U02, INU (top), 7 foreign binders AH1, XV6, AHF, 1UN, A88, 846, BH0 (middle), and 11 non-binder molecules ARA, CEL, GAL, LSN, OLM, SAM, ZAF, ZED, ADN, 107, and U55 (bottom).**
(TIFF)

Table S1 Results of “leave-correct-receptor-structure-out” tests.
(DOC)

Table S2 Best RMSD and top-scoring results of true binder docking.
(DOC)
