# Supplementary figures and images for: ReFlexIn: A Flexible Receptor Protein-Ligand Docking Scheme Evaluated on HIV-1 Protease
Source: PLoS One. 2012 Oct 24;7(10):e48008. doi: 10.1371/journal.pone.0048008 (PMC3480487; doi:10.1371/journal.pone.0048008)

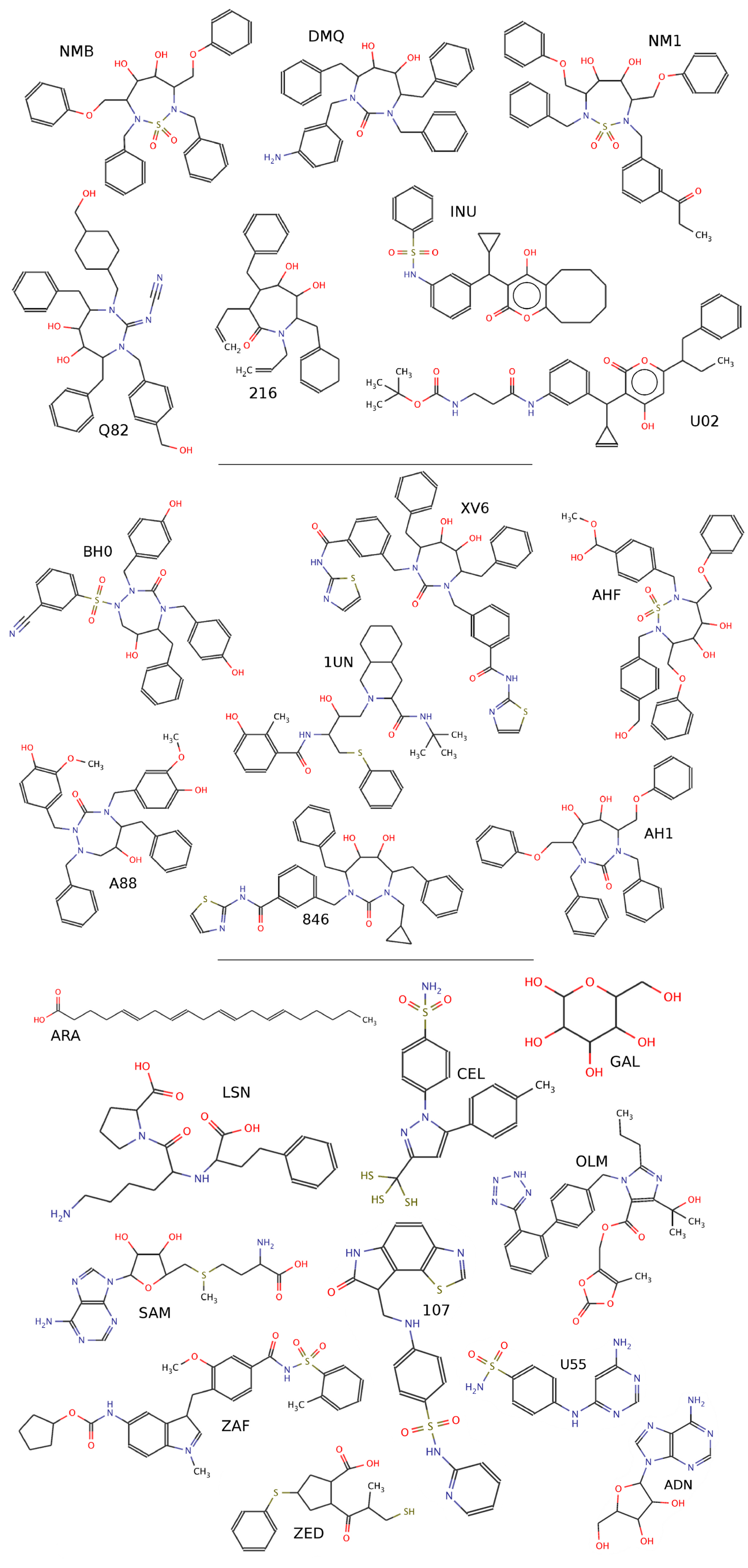

Supplement: Figure S1 — Full ligand test-set containing 7 true binders NMB, DMQ, NM1, Q82, 216, U02, INU (top), 7 foreign binders AH1, XV6, AHF, 1UN, A88, 846, BH0 (middle), and 11 non-binder molecules ARA, CEL, GAL, LSN, OLM, SAM, ZAF, ZED, ADN, 107, and U55 (bottom). (TIFF) [file pone.0048008.s001.tiff]
