# Supplementary material for: ReFlexIn: A Flexible Receptor Protein-Ligand Docking Scheme Evaluated on HIV-1 Protease
Source: PLoS One. 2012 Oct 24;7(10):e48008. doi: 10.1371/journal.pone.0048008 (PMC3480487; doi:10.1371/journal.pone.0048008)
Supplement: Table S1 — Results of “leave-correct-receptor-structure-out” tests. (DOC) [file pone.0048008.s002.doc]

**Table S1. Results of “leave-correct-receptor-structure-out” tests**

|  | ligand rigid | | | ligand flexible | | |
| --- | --- | --- | --- | --- | --- | --- |
|  | bound-all | bound-csm | apo | bound-all | bound-csm | apo |
| NMB | 100 | 100 | 0 | 63 | 44 | 0 |
| DMQ | 100 | 100 | 0 | 99 | 99 | 0 |
| NM1 | 100 | 99 | 19 | 75 | 83 | 0 |
| Q82 | 60 | 96 | 0 | 0 | 1 | 0 |
| 216 | 100 | 100 | 0 | 100 | 98 | 0 |
| U02 | 62 | 0 | 0 | 38 | 22 | 0 |
| INU | 84 | 84 | 0 | 60 | 40 | 0 |

Percentages of docking results with an RMSD_ligand_ < 2.0 Å when comparing the flexible receptor dockings with all bound HIV1-Protases structures used (bound-all) and using all bound structures except the partner structure of the docked ligand (bound-csm, csm stands for 'correct structure missing')
