# Supplementary material for: ReFlexIn: A Flexible Receptor Protein-Ligand Docking Scheme Evaluated on HIV-1 Protease
Source: PLoS One. 2012 Oct 24;7(10):e48008. doi: 10.1371/journal.pone.0048008 (PMC3480487; doi:10.1371/journal.pone.0048008)
Supplement: Table S2 — Best RMSD and top-scoring results of true binder docking. (DOC) [file pone.0048008.s003.doc]

**Table S2.** **Best RMSD and top-scoring results of true binder docking**

|  | ligand rigid | | | | ligand flexible | | | |
| --- | --- | --- | --- | --- | --- | --- | --- | --- |
|  | Flexible Receptor Docking | | Apo Docking | | Flexible Receptor Docking | | Apo Docking | |
|  | best RMSD (Å) | top-score RMSD (Å) | best RMSD (Å) | top-score RMSD (Å) | best RMSD (Å) | top-score RMSD (Å) | best RMSD (Å) | top-score RMSD (Å) |
| NMB | 0.40 | 0.42 | 7.54 | 7.74 | 0.40 | 0.40 | 2.09 | 7.58 |
| DMQ | 0.48 | 0.51 | 7.73 | 7.74 | 0.38 | 0.42 | 6.14 | 7.87 |
| NM1 | 0.34 | 0.35 | 1.27 | 10.34 | 0.37 | 0.42 | 6.45 | 7.38 |
| Q82 | 1.74 | 5.50 | 5.40 | 5.59 | 2.74 | 5.60 | 5.42 | 5.97 |
| 216 | 0.48 | 0.48 | 6.30 | 6.45 | 0.59 | 0.71 | 2.10 | 6.73 |
| U02 | 0.74 | 1.08 | 9.82 | 9.86 | 0.68 | 0.99 | 5.57 | 9.23 |
| INU | 0.54 | 0.60 | 9.06 | 9.07 | 0.68 | 0.83 | 6.02 | 6.50 |

Best RMSD (i.e. the lowest yielded RMSD_ligand_ out of 100 separate docking runs) and the RMSD values of the top-scoring docking solutions for different ligands and receptor flexibility.
